# Semi-supervised learning of causal relations in biomedical scientific discourse

**DOI:** 10.1186/1475-925X-13-S2-S1

**Published:** 2014-12-11

**Authors:** Claudiu Mihăilă, Sophia Ananiadou

**Affiliations:** 1 The National Centre for Text Mining, The University of Manchester, 131 Princess Street, M1 7DN Manchester, UK

**Keywords:** text mining, discourse analysis, biomedical causality

## Abstract

**Background:**

The increasing number of daily published articles in the biomedical domain has become too large for humans to handle on their own. As a result, bio-text mining technologies have been developed to improve their workload by automatically analysing the text and extracting important knowledge. Specific bio-entities, bio-events between these and facts can now be recognised with sufficient accuracy and are widely used by biomedical researchers. However, understanding how the extracted facts are connected in text is an extremely difficult task, which cannot be easily tackled by machinery.

**Results:**

In this article, we describe our method to recognise causal triggers and their arguments in biomedical scientific discourse. We introduce new features and show that a self-learning approach improves the performance obtained by supervised machine learners to 83.47% for causal triggers. Furthermore, the spans of causal arguments can be recognised to a slightly higher level that by using supervised or rule-based methods that have been employed before.

**Conclusion:**

Exploiting the large amount of unlabelled data that is already available can help improve the performance of recognising causal discourse relations in the biomedical domain. This improvement will further benefit the development of multiple tasks, such as hypothesis generation for experimental laboratories, contradiction detection, and the creation of causal networks.

## Background

With the advent of online publishing of scientific research came an avalanche of electronic resources and repositories containing knowledge encoded in some form or another. In the domain of biomedical sciences, research is now being published at a faster-than-ever pace, with several thousand articles per day. It is impossible for any human being to process that amount of information in due time, let alone apply it to their own needs. Thus appeared the necessity of being able to automatically retrieve relevant documents and extract useful information from text. Significant advances have been made towards biomedicine-specific tasks such as recognising named entities [1], relations and events between them [2], but also towards NLP-oriented tasks such as coreference resolution [3] and automatic summarisation [4,5]. With the help of text mining, biomedical researchers can now easily query the vast databases for articles of interest and, moreover, obtain important information without searching manually. The text can be processed easily and researchers have the freedom to customise the processing according to their specific requirements using workflow-building platforms, such as U-Compare [6] and Argo [7]. Furthermore, the published information can be automatically organised into meaningful structures, such as metabolic and signalling pathways [8].

Although it is now possible to distil essential factual knowledge from text, it is difficult to interpret the connections between extracted facts. These connections, also known as *discourse relations*, make the text coherent and cohesive, and their automatic discovery can lead to a better understanding of the conveyed knowledge. They can be either explicit or implicit, depending on whether or not they are expressed in text using overt *discourse connectives *(also known as *triggers*). One of the fundamental discourse relations is causality, as it explains the functioning of ourselves, our environment and our interaction with it. But causal relations pose two main difficulties when trying to recognise them, one regarding causal triggers, and the other regarding their arguments.

First, causal triggers are both highly ambiguous and highly variable. Take, for instance, the following example, where the token *and *expresses causality. However, in most other contexts, the same token has a non-causal meaning. The conjunction *and *occurs only once with a causal meaning in the BioCause corpus [9], which is much less than the number of non-causal instances (2305).

(1) SsrB binds within SPI-2 *and *activates SPI-2 genes for transcription.

This is the usual case with most closed-class part-of-speech words, such as conjunctions and adverbials. Other examples of trigger types more commonly used as causal triggers and belonging to open-class parts-of-speech are *suggesting *(9 causal instances, 54 non-causal instances), *indicating *(8 causal instances, 41 non-causal instances) and *resulting in *(6 causal instances, 14 non-causal instances). For instance, example (2) contains two mentions of *indicating*, but neither of them implies discourse causality.

(2) Buffer treated control cells showed intense green staining with syto9 (*indicating *viability) and a lack of PI staining (*indicating *no dead/dying cells or DNA release).

Furthermore, their variability results in numerous ways of expressing the same causal trigger, due to the open-class properties of nouns and verbs. Take example (3), where the trigger *this result suggests that *indicates a causal relation.

(3) The hilE mRNA level measured by real-time PCR also revealed that hilE expression was increased in SR1304 by about 2-fold (Figure 3A). *This result suggests that *Mlc can act as a negative regulator of hilE.

The same idea can be conveyed using synonyms of these words, such as *observation, experiment, indicate, show, prove *etc. The high variability reflects in obtaining a low recall, since there will be many false negatives (FNs).

With respect to the two arguments, they are more difficult to recognise than causal triggers. First, the spans of text that make up the arguments are of arbitrary length, varying significantly from one case to another, as previously reported by Mihăilă et al. [9]. Arguments can go up to 100 tokens in length in the case of Cause, and up to 70 in the case of Effect.

Second, the position of the two arguments around the trigger can change. Although most of the relations follow a Cause-Trigger-Effect pattern, there is an important percentage of relations, 20%, which do not obey this rule. Furthermore, Mihăilă et al. [9] show that almost half of all relations have one argument in a different sentence than that of the trigger. Thus, the search space increases significantly and the difficulty of a correct recognition increases too.

This leads to the third reason, which regards the distance between the trigger and the arguments. Mihăilă et al. [9] illustrate the number of sentences between that of the trigger and that of the independent argument, when it is located in a different sentence. About half of the cases have the argument located in the previous sentence, but the rest spread up to the tenth previous sentence.

In order to automate this process, human experts have developed manually annotated corpora, such as the Penn Discourse Treebank (PDTB) [10], a corpus of lexically-grounded annotations of discourse relations in the general domain. Based on this corpus, researchers have not only identified discourse connectives, but also developed end-to-end discourse parsers [11,12]. However, biomedical discourse has been shown to exhibit different traits when compared to general language, at multiple levels. Be it lexical, syntactic, semantic or discourse-level, biomedical researchers use a different language to convey information [13,14]. As an effect, automatic systems trained on general language might not work as well when applied to biomedical text. Yet, comparatively little work has been carried out on causal discourse relations in the biomedical domain, although causal associations between biological entities, events and processes are central to most claims of interest [15].

The equivalent of the PDTB for the biomedical domain is the BioDRB corpus [16], containing 16 types of discourse relations, e.g., temporal, causal and conditional. The number of purely causal relations annotated in this corpus is 542. A slightly larger corpus is BioCause [9], containing over 850 manually annotated causal discourse relations in 19 full-text open-access journal articles from the infectious diseases domain. Out of these, 800 relations are explicit, meaning that the trigger is overtly expressed in the text.

Using the BioDRB corpus as data, some researchers explored the identification of discourse connectives [17,18]. However, they do not distinguish between the types of various discourse relations. Ramesh et al. [17] obtain the best F-score of 75.7% using conditional random fields (CRFs), whilst Ibn Faiz et al. [18] reach 82.36% F-score using a maximum entropy (ME) classifier. These results were obtained by using only syntactic features, as semantic features were shown to lower the performance. Also, Ramesh et al. [17] prove that there exist differences in discourse triggers between the biomedical and general domains by training a model on the BioDRB and evaluating it against PDTB and vice-versa.

Mihăilă et al. [19] focus on causal triggers only. With experiments on BioCause, triggers are recognised with 79% F-score by employing CRFs. They use a wide array of features, including lexical, syntactic and semantic information.

In this paper, we describe our attempt to overcome the issue of the little amount of available gold standard annotations for causality in biomedical discourse. We do this by using both labelled and unlabelled data in a semi-supervised learning (SSL) framework, where a classifier learns by itself from a large unlabelled dataset based on a small labelled corpus, increasing the confidence of its decisions in the process. We show that this method improves the performance obtained by existing approaches based only on gold standard data. Moreover, we add novel structural features that reduce the number of false negatives generated by the highly skewed corpora.

## Methods

This section describes the data used for the experiments, as well as the feature set and self-learning algorithm.

### Data

The data for the experiments comes from the BioCause corpus, a collection of 19 open-access full-text journal articles pertaining to the biomedical subdomain of infectious diseases, manually annotated with 850 causal relationships. Two types of spans of text are marked in the text, namely causal triggers and causal arguments. Each causal relation is composed of three text-bound annotations: a trigger, a cause argument and an effect argument. Some causal relations have implicit triggers, but these are excluded from the current research as their number is very small (more specifically, 50).

**Figure 1:**
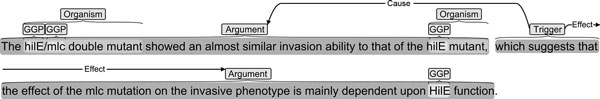
**Causal relation in BioCause**. Causal relation as annotated in the BioCause corpus, with marked trigger and its two arguments, as well as named entities.

Figure 1 shows an example of discourse causality from BioCause, marking the causal trigger and the two arguments with their respective relation. Named entities are also marked in this example.

BioCause contains 381 unique explicit triggers, each being used, on average, only 2.10 times. The number decreases to 347 unique triggers when they are lemmatised, corresponding to an average usage of 2.30 times per trigger. Both count settings demonstrate the diversity of causality-triggering phrases that are used in the biomedical domain.

The unlabelled data consists of 50 full-text open-access journal articles also on infectious diseases of similar age as those in BioCause. These conditions have been imposed with the knowledge that biomedical subdomains differ in terms of the semantic types they include [14]. However, unlike BioCause, they do not contain any type of gold standard annotations. All features that are used in the experiments, described in what follows, are derived from fully automatic parses.

### Pipeline

**Figure 2:**
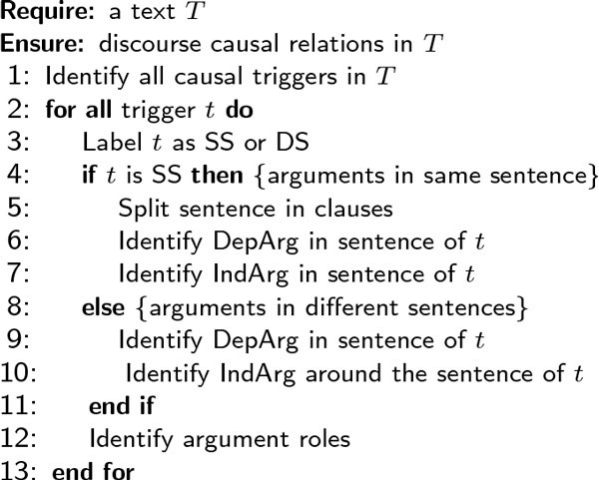
**Causal relation identification pseudocode**. Pseudocode for identifying causal relations in the BioCause.

The pseudocode for the causality recognition pipeline is shown in Figure 2. Similar to the annotation mechanism used by the experts who produced the BioCause corpus, we have split the recognition of causality into three major steps. First, the annotators were given just the raw text *T *, which was then analysed to find causal triggers. We modelled trigger span detection (TS) both as a classification task, using Support Vector Machines (LibSVM [20]) and Random Forests (Weka [21]), and as a sequence labelling task, using CRF [22] (CRFSuite). Second, when a causal trigger was found, the annotators decided on the argument position (AP), i.e. whether its two arguments are in the same sentence (SS) or different sentences (DS). In the former case, the clause syntactically depending on the trigger becomes the dependent argument (DepArg), whilst the rest of the sentence represents the independent argument (IndArg). In the latter case, the sentence containing the trigger becomes the dependent argument, whilst the independent argument is identified as one of the sentences around the trigger. We used different machine learners to distinguish between intra- and inter-sentential relations and to detect the argument spans (AS) for each case. Finally, in the third step, after both arguments are located, the annotator classifies the direction of the relation, that is which argument plays which of the semantic roles of cause and effect (AR). We trained several models to assign roles to the previously detected arguments.

### Features

Feature engineering and selection is a vital part of any machine learning system. Various types of features have previously been used for the task of detecting causal triggers and their arguments, including lexical, syntactic, semantic and statistical (bag of words) features. However, most past work has concentrated around lexical and syntactic features, whilst the semantic aspects of causality (like named entities and events) have been ignored or deemed detrimental to the task in the few cases in which they were considered [17]. In addition to these features, we introduce a new set of features derived from command relationships and position in sentence.

Thus, based on our analysis of causal triggers, we engineered six types of features for the development of this causality model, i.e., lexical, syntactic, dependency, command, semantic and position in sentence. A more detailed description is given in subsequent sections. For each feature, we specify in which of the four step of the pipeline it is used (TS, AP, AS or AR).

#### Lexical features

The lexical features are built from the actual tokens present in text, and are summarised in Table 1. Their utility has been noticed by several researchers [23,12,18], who state that both the surface level token and its neighbours help towards a correct classification.

**Table 1:** Lexical features used in identifying causal relations.

ID	Short description	T	AP	AS	AR
L1	token	✓	✓	✓	✓
L2	*lemma*(token)	✓	✓	✓	✓
L3	*neighbour *(token,[left, right],[1..5])	✓	✓	✓	✓
L4	*lemma*(*L3 *)	✓	✓	✓	✓
L5	*isCapitalised *(trigger)		✓	✓	✓

The tokenisation and lemmatisation steps are performed by employing the GENIA tagger [24] trained on MEDLINE. The first two features represent the token's surface expression and its lemma. The inclusion of lemmata is justified by the need of generalisation: some inflected lexemes may occur very rarely (if at all) in the limited amount of training data, and, in a real-world deployment, a learner may be perplexed when encountering them.

On the other hand, there exists a need for specialisation due to the polysemy and homonymy of words. The context can affect the meaning of a token and therefore it is necessary to include surrounding tokens in order to allow a learner to differentiate between, for instance, *and *as a causal trigger or enumerating conjunction. Thus, we included the five tokens immediately to the left and the ones immediately to the right of the current token. In the case of causal triggers, this decision is based on two observations. First, in the case of tokens to the left, most triggers are found either at the beginning of the sentence (311 instances) or are preceded by a comma (238 instances). These two left contexts represent 69% of all triggers. Second, for the tokens to the right, almost 45% of triggers are followed by a determiner, such as *the, a *or *an *(281 instances), or a comma (71 instances).

In the case of arguments, when the trigger is the token *Thus *(i.e., *thus *with a capital first letter), it is highly probable that the current sentence is an effect of a previous sentence. Furthermore, a useful feature is a flag saying whether the trigger starts with a capital letter or not, L5. This again helps in the decision for the position of the trigger in the sentence.

#### Syntactic features

Syntax is the main provider of features in the literature. Almost all approaches use the part-of-speech and syntactic category of the token and its neighbours [11,23,17,18]. Pitler et al. [11] explores the parse tree horizontally, including the neighbours into the equation. In contrast, Wellner [23] explores it vertically, deriving features from the path from the root of the parse tree to the token.

**Figure 3:**
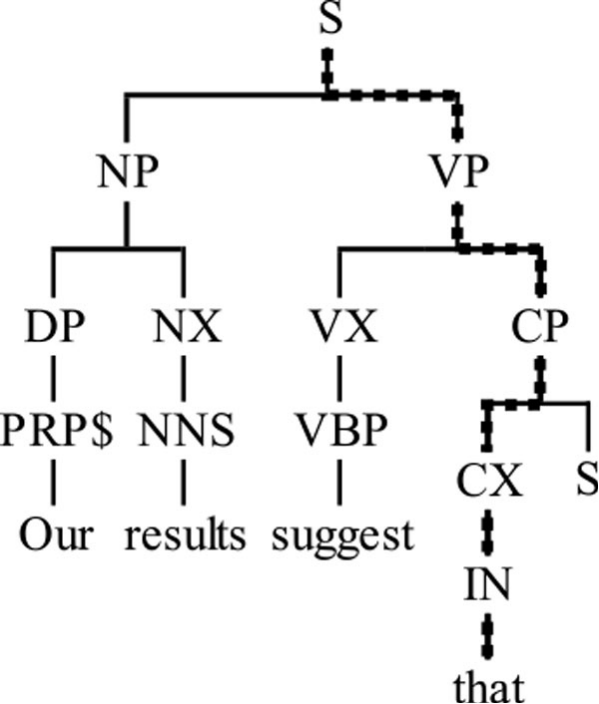
**Partial lexical parse tree**. Partial lexical parse tree of a sentence starting with a causal trigger.

The syntax, dependency and predicate argument structure are produced by the Enju parser [25]. Figure 3 depicts a partial lexical parse tree of a sentence which starts with a causal trigger, namely *Our results suggest that*. From the lexical parse trees, several types of features have been generated, a list of which is included in Table 2.

**Table 2:** Syntactic features used in identifying causal relations.

ID	Short description	T	AP	AS	AR
X1	*partOfSpeech*(token)	✓		✓	
X2	*syntCat*(token)	✓		✓	
X3	*partOfSpeech*(*L3*)	✓		✓	
X4	*syntCat*(*L3*)	✓		✓	
X5	*syntCatPathFromRoot*(token)	✓			
X6	*syntCatCollapsedPathFromRoot*(token)	✓			
X7	*syntCatPositionPathFromRoot*(token)	✓			
X8	*ancestor *(token,[1..3])	✓			
X9	*lowestCommonAncestor *(token,*neighbourOf *(token,left,1))	✓			
X10	*distanceBetween*(token, *X9*)	✓			
X11	*posString *(trigger)		✓		✓
X12	*syntCatString *(trigger)		✓		✓
X13	*posStringDupl *(trigger)		✓		✓
X14	*syntCatStringDupl *(sent)		✓		✓
X15	*containsMainVerb*(trigger)		✓		✓
X16	*mainVerb*(sent)			✓	
X17	*voiceOfVerb*(trigger)				✓

The first two features represent the part-of-speech and syntactic category of a token. For instance, the figure shows that the token *that *has the part-of-speech *IN*, whilst its syntactic category is *P*. These features are included due to the fact that either many triggers are lexicalised as an adverb or conjunction, or are part of a verb phrase.

For the same reason, the syntactical category path from the root of the lexical parse tree to the token is also included as X5. Because in parse trees there are many cases where constituents will repeat when moving vertically, we collapse X5 into a new feature (X6) by deleting consecutive repetitions of the same syntactic category. For instance, in a path such as *S/VP/VP/V*, the adjacent identical tags *VP/VP *are combined into *VP*, thus creating a collapsed path of *S/VP/V*.

Also based on X5, the path encodes in feature X7, for each parent constituent, the position of the token in its subtree, i.e., beginning (*B *), inside (*I *) or end (*E *); if the token is the only leaf node of the constituent, this is marked differently, using a *C*. Thus, the path of *that*, highlighted in the figure, is *I-S/I-VP/B-CP/C-CX*. Feature X7 has been used before by Ghosh et al. [26], whilst Wellner et al. [27] used X5, both in their task of extracting the arguments of discourse triggers in general.

Furthermore, the ancestors of each token to the third degree are instantiated as three different features. This has been found by Ibn Faiz et al. [18] to better generalise the syntactic context of the token than X5, although they restrict it to only the first parent. In the case that such ancestors do not exist (i.e., the root of the lexical parse tree is less than three nodes away), a "none" value is given. For instance, the token *that *in Figure 3 has as its first three ancestors the constituents marked with *CX, CP *and *VP*.

Finally, the lowest common ancestor in the lexical parse tree between the current token and its left neighbour has been included. The lowest common ancestor of two nodes A and B in a dependency tree is a node L, and there exists no other node N such that L is an ancestor of N. In the previous tree example in Figure 3, the lowest common ancestor for *that *and *suggest *is *VP*.

The following two feature types have been produced on the observation that the lowest common ancestor for all tokens in a causal trigger is S or VP in over 70% of instances. Furthermore, the percentage of cases of triggers with V or ADV as lowest common ancestor is almost 9% in each case. Also, the average distance to the lowest common ancestor is 3.

We include PoS and syntactic category strings representations of the causal triggers (X11 and X12, respectively). For instance, a trigger such as *These results show that *is represented as a PoS string *DT-NN-V-DT*. This adds a level of generalisation, where (usually) nouns and verbs can be replaced by their numerous synonyms.

These two features are then extended by creating other strings which do not contain duplicate consecutive PoS or syntactic category values, marked as X13 and X14. In other words, *DT-NN-V-V-DT *is reduced to *DT-NN-V-DT*. This simplifies the string representation and reduces the data sparsity. A sequence of adjectives or compound verb tenses should not affect the causal relation.

We also add a feature, X15, indicating whether the trigger contains the sentence's main verb. If it does, this is a good indicator that the arguments are located in different sentences. Furthermore, feature X16 contains the main verb of the sentence. We are also interested in the voice of the verb, which is included as feature X17. This is helpful in determining the direction of the relation: which predicate affects which?

#### Dependency features

These features are constructed based on the dependency relations found by Enju in the sentence. Table 3 includes all dependency features employed in this study.

**Table 3:** Dependency features used in identifying causal relations.

ID	Short description	T	AP	AS	AR
D1	*pas*(token)	✓		✓	
D1	*pas-role*(token)	✓		✓	
D2	*pos*(D1)	✓		✓	
D3	*distanceBetween*(token,D1)	✓		✓	

First, for each token, we extracted the predicate-argument structures and included the arguments as surface expression forms. We also included the parts-of-speech of these arguments, as well as the distance from the token.

#### Constituency features

Command features are constructed from *command relations *found in the constituency parse tree of the sentence. The concept of command relation was initially introduced by Langacker et al. [28], who defined it as "a node X commands a node Y if neither X nor Y dominates the other and the S (sentence) node most immediately dominating X also dominates Y". A more general definition has been provided by Reinhart [29], who defined a *constituent command *(*c-command *) by eliminating the restriction of having the node dominating both X and Y being a sentence. Barker et al. [30] relaxed this definition even further, by removing the non-co-dominance condition between X and Y.

**Figure 4:**
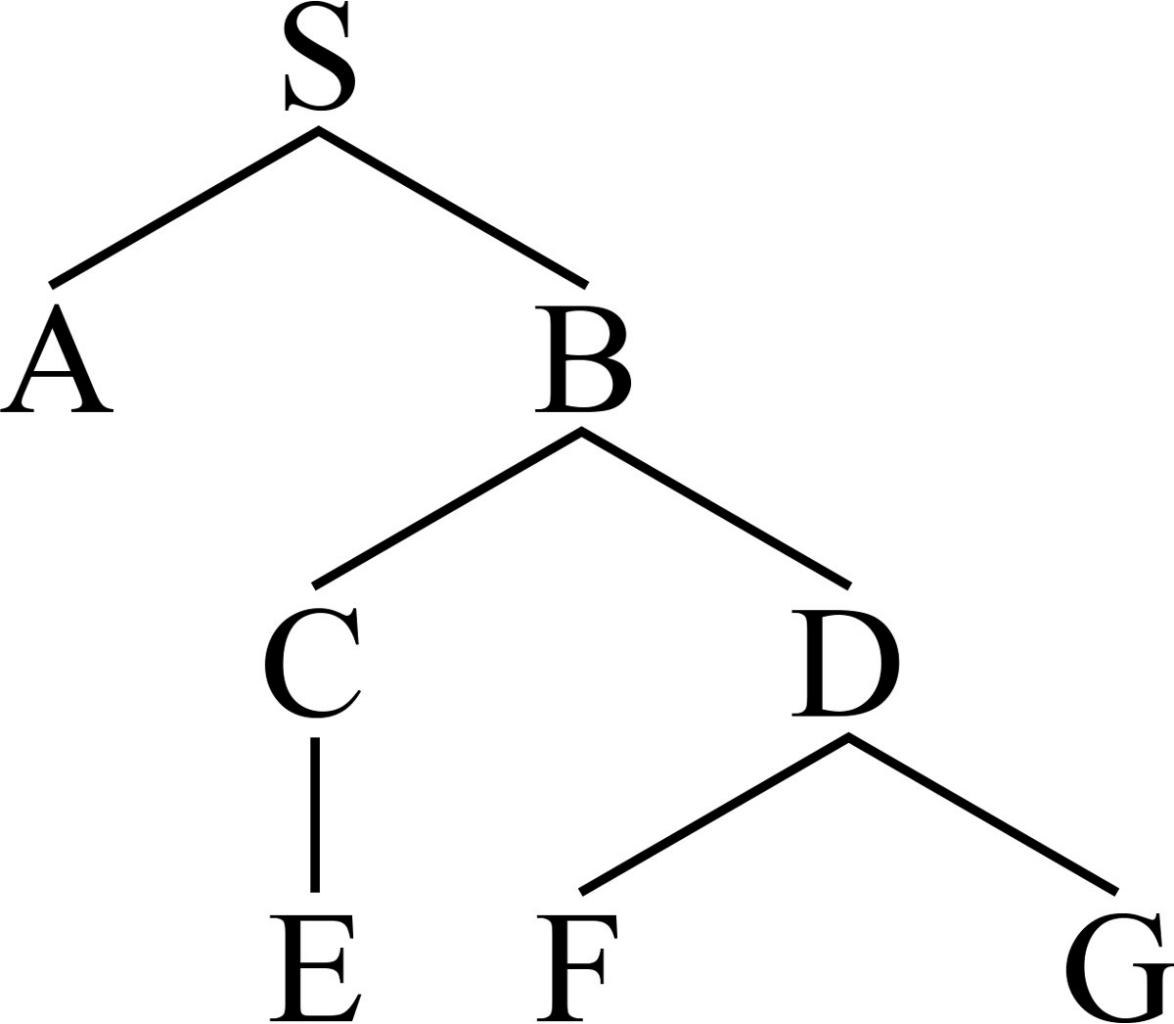
**c-command syntax tree**. c-command syntax tree: A c-commands B, B c-commands A, C c-commands D, D c-commands C etc.

Based on command relations as defined by Barker et al. and exemplified in Figure 4, we developed several features, which, to the best of our knowledge, have not been previously used for identifying discourse causal triggers and arguments. These are included in Table 4.

**Table 4:** Constituency features used in identifying causal relations.

ID	Short description	T	AP	AS	AR
C1	*c-commands*(token, SBAR)	✓		✓	
C2	*c-commands*(token, VP)	✓		✓	
C3	*c-commands*(token, NP)	✓		✓	
C4	*S-commands*(token, SBAR)	✓		✓	
C5	*S-commands*(token, VP)	✓		✓	
C6	*S-commands*(token, NP)	✓		✓	
C7	*VP-commands*(token, SBAR)	✓		✓	
C8	*VP-commands*(token, VP)	✓		✓	
C9	*VP-commands*(token, NP)	✓		✓	

Features C1-C3 indicate whether the current token c-commands a SBAR, VP or NP constituent, respectively. Features C4-C6 are similar, with the exception that the dominant node must be an S (sentence). In the case of features C7-C9, the dominant node must be a VP.

All mentioned features rely on the observation that a trigger c-commands at least one of its arguments (more specifically, the dependent argument). In most cases, trigger tokens S-command or VP-command argument tokens, whose superparent is usually an SBAR, VP, or NP.

#### Semantic features

Although the role of semantic features has been previously explored, the results are contradictory. In one study in the biomedical domain, adding a semantic layer lowers the performance of recognising discourse triggers [17], whilst in the general domain rich compositional semantic information (i.e. VerbNet and CoreLex) manages to produce a statistically significant increase in F-score [31]. Ramesh et al. [17] use the BANNER gene tagger and LINNAEUS species tagger to obtain named entity information about genes and species, as well as Metamap to map text elements to UMLS.

We have exploited several semantic knowledge sources to identify causal triggers and arguments more accurately, as a mapping to concepts, named entities and events acts as a back-off smoothing, thus increasing performance. This happens due to the fact that causal triggers do not encode biomedical knowledge, thus tokens recognised as named entities or events should not be recognised as causal triggers, whilst arguments should contain biomedical semantics. A list of all semantic features in included in Table 5.

**Table 5:** Semantic features used in identifying causal relations.

ID	Short description	T	AP	AS	AR
S1	*isNamedEntity *(token)	✓		✓	
S2	*namedEntityType*(token)	✓		✓	
S3	*isEvent*(token)	✓		✓	
S4	*eventType*(token)	✓		✓	
S5	*wordnetHypernym*(token)	✓		✓	
S6	*isUMLSEntity *(token)	✓		✓	
S7	*UMLSEntityType*(token)	✓		✓	
S8	*isTrigger *(token)			✓	
S9	*isDA*(token)			✓	

One semantic knowledge source is the BioCause corpus itself. All documents annotated for causality in BioCause had been previously manually annotated with biomedical named entity and event information. This was performed in the context of various shared tasks, such as the BioNLP 2011 Shared Task on Infectious Diseases [32]. We therefore leverage this existing information to add another semantic layer to the model. Moreover, another advantage of having a gold standard annotation is the fact that it is now possible to separate the task of automatic causal trigger recognition from automatic named entity recognition and event extraction. The named entity and event annotation in the BioCause corpus is used to extract information about whether a token is part of a named entity or event trigger. Furthermore, the type of the named entity or event is included as a separate feature. Whilst named entities have been employed before [17], to the best of our knowledge, event information has not.

The second semantic knowledge source is WordNet [33]. Using this resource, the hypernym of every token in the text has been included as a feature. This is needed for those tokens which are not specific to biomedicine. Only the first sense of every token has been considered, as no sense disambiguation technique has been employed. Finally, tokens have been linked to the UMLS [34] semantic types. Thus, we included a feature to say whether a token is part of a UMLS type (S6) and another for its semantic type if S6 is true.

The other two features, S8 and S9, record the decisions made by the systems in previous steps. For instance, feature S8 is used in the second and third step of our argument detection pipeline, and shows whether or not a token has been marked as a trigger. Similarly, S9 is used only in the last step and shows whether or not a token has been marked as being part of the dependent argument.

#### Positional features

Position features have also been engineered and included in Table 6. First, the location of the token in the sentence is important, as most of the triggers occur in the beginning or middle of the sentence. On the other hand, the position of the trigger in the sentence is also of great importance. An initial trigger suggests that the arguments are located in different sentences, whilst a trigger in mid-sentence tends to have both arguments around it in the same sentence. This feature takes integer values, representing the index in the sentence. However, due to the various sentence lengths in which causality occurs, this may result in data sparseness. Thus, we add a feature which shows the token's index in the sentence percentage-wise. That is, we divide the value of feature P1 by the length of the sentence. To be more discrete, we also add a feature which takes only three values: "Beginning", "Middle", and "End". Furthermore, the sentence length has been included, as this is correlated with the position: the shorter the sentence, the smaller the chances that a token is part of a trigger in the middle of the sentence.

**Table 6:** Positional features used in identifying causal relations.

ID	Short description	T	AP	AS	AR
P1	*indexInSent*(token)	✓	✓	✓	✓
P2	*percentageInSent*(token)	✓	✓	✓	✓
P3	*positionInSent*(token)	✓	✓	✓	✓
P4	*length*(*sentence*(token))	✓	✓	✓	✓

### Semi-supervised learning

In a semi-supervised learning setting, we modelled the problem as a self-training task. The main reason for including this method is the limited amount of existing gold standard data. Self-training has been previously used in NLP applications, such as word sense disambiguation [35], identification of subjective nouns [36] and emotions in dialogues [37]. Nevertheless, to the best of our knowledge, it has not been applied in discourse (causal) relation recognition.

**Figure 5:**
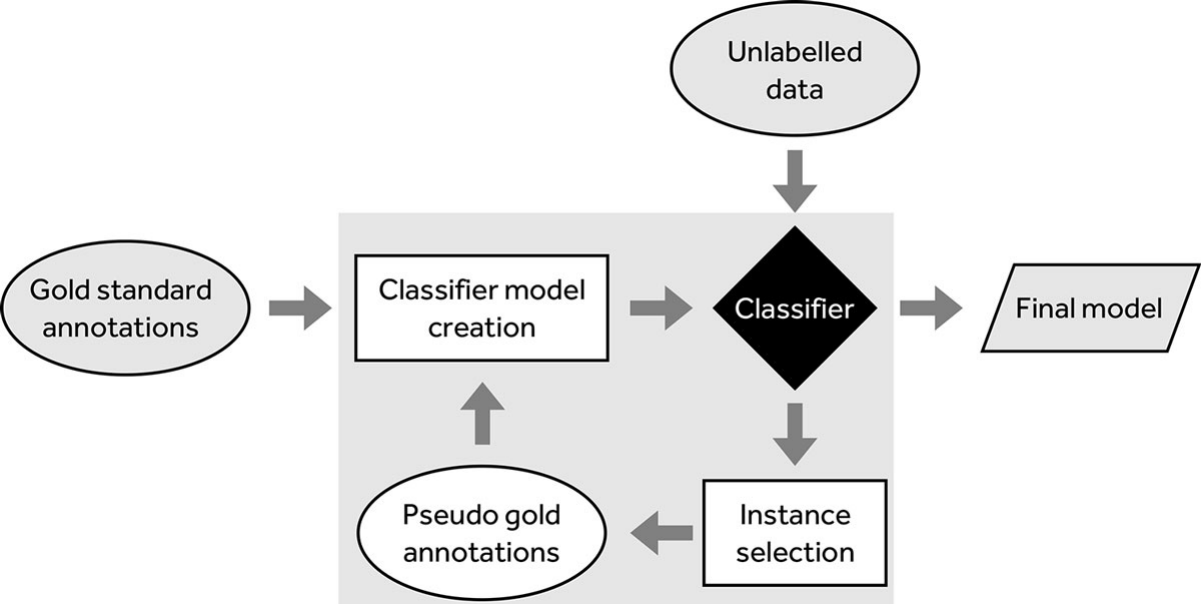
**Self training approach**. The process of self training.

The entire learning process is depicted visually in Figure 5. We have started the learning process with a small amount of labelled data, Λ, for classifier training. This results in the creation of a classification model, *µ*. Then, the unlabelled data, Υ, is classified using *µ*. From these newly obtained classifications, only those instances that have a classification confidence higher than a pre-set threshold *τ *are considered gold and are added to the labelled data as classified by *µ*. The rest are kept as unlabelled. If there are no instances that are classified with a confidence greater than *τ *, the model would come to a blocked state. Thus, we apply some simple heuristics to select several instances to be added to the labelled data. The process is repeated until all instances are classified.

In this case, the gold standard data is represented by BioCause. We have split BioCause into two equally sized sets of 400 causal relations each. One set is used for the seed set, whilst the other is used for the final model evaluation. The experiment is then repeated with swapped sets, and the results are averaged. Although the seed and test sets are not very large in size, we believe that they can be used to prove the validity of the method. Of course, evaluation and validation on larger corpora is necessary, but these datasets still need to be created.

## Results

We trained models with different sizes for the seed labelled sets Λ. There are eight models, varying in the percentage of positive instances from 12.5% to 100%, in steps of 12.5%, extracted from the self-training part of the corpus. On the other hand, we changed the ratio of positive to negative instances in each labelled set. The ratios are 1:1, 1:2, 1:5, and the actual ratio in BioCause, approximately 1:50. In creating these subsets, we use all positive instances available, and then randomly choose the corresponding number of negative instances.

### Trigger detection

For the supervised classification part of SSL, we have employed CRFs, RFs and SVMs, as they have performed best in the experiments described in previous research [38]. As for the heuristics used in case no instance is classified with a confidence greater than *τ *, we have used several rule-based routines. We consider for marking as labelled instances only those which have the confidence in the top 5% of all confidences. We then filter these instances and select only those which have several feature values that were deemed important by automatic attribute evaluators, i.e. InfoGain and ChiSquare. These include the lemma of the token (L2), the predicate-argument structure links of the token and ancestor constituents (D1, D2), its c-command and VP-command values (C1-C3, C7-C9), and named entity information (S1, S5, S6). The lemma has to be part of a lexicon of lemmas contained in causal triggers that is pre-compiled. At least one of the ancestor constituents must be either a VP, NP or S. The token must c-command or VP-command a VP or NP. Furthermore, the token must not bear any biomedical meaning. These rules are given equal weights, and each token must comply with at least two of the rules in order to be considered as labelled correctly.

**Figure 6:**
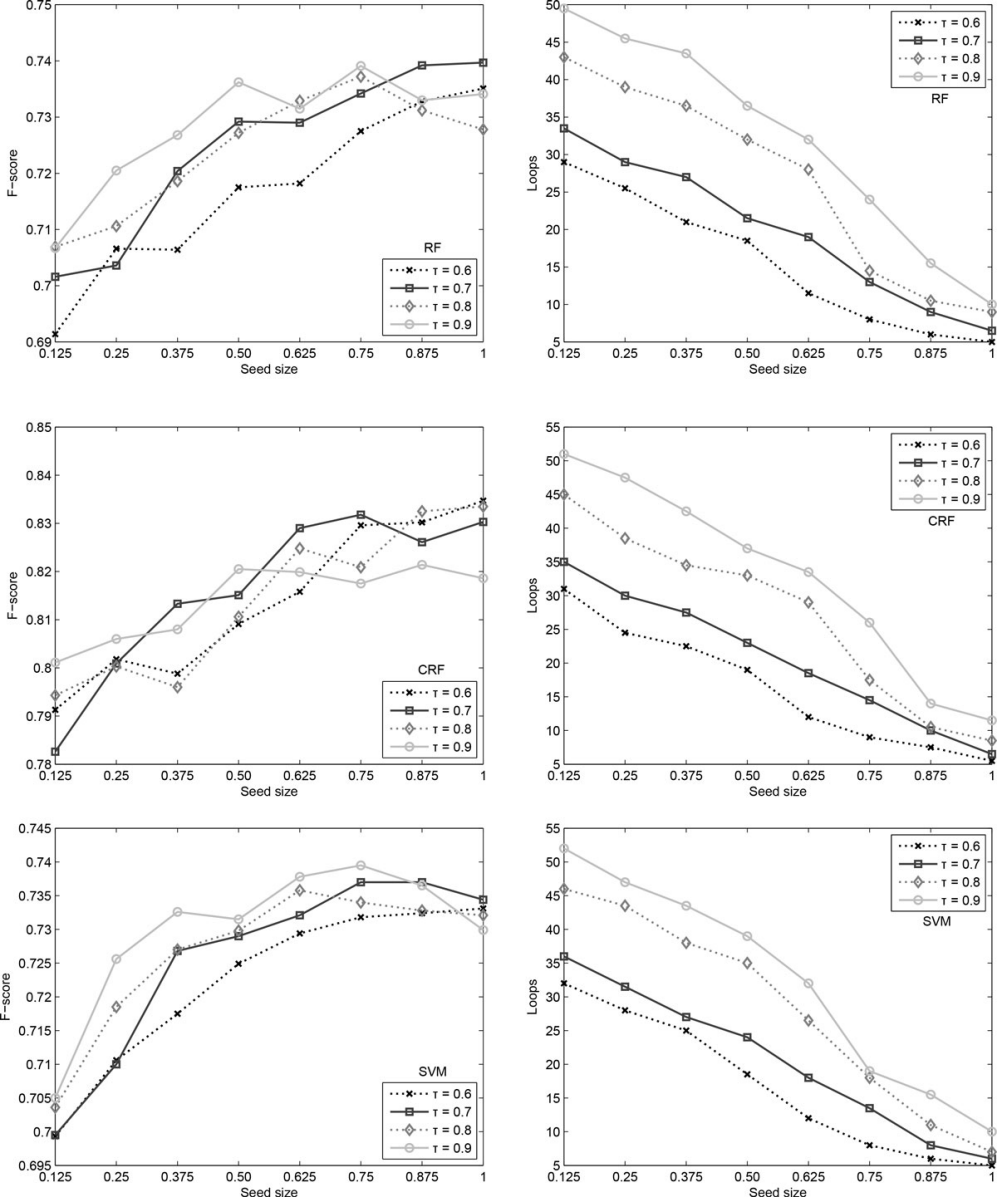
**Trigger detection results and loops needed**. Self-training results (left) and number of self-training loops (right) when varying *τ *for the natural ratio in trigger detection.

The results are summarised in Table 7 together with the top performance reported by Mihăilă et al. [38]. Figure 6 shows the performance of the three classifiers when varying *τ *from 0.6 to 0.9 confidence and the seed size from 12.5% to 100%, whilst keeping the natural ratio of positive:negative instances. As can be noticed, all models have a generally increasing trend, showing that the amount of gold standard training data is essential to this task. Furthermore, the learning curve does not turn into a plateau when a high percentage of data is available for training. This suggests that the performance could be improved if more data were available. The top results, when the seed size is 100%, are slightly higher than those obtained by employing supervised algorithms.

**Table 7:** Performance of various semi-supervised classifiers in identifying trigger spans.

Classifier	P	R	F_1_
Mihăilă et al. [38]	89.00%	74.00%	79.00%

CRF	86.34%	80.56%	83.35%
SVM	82.45%	66.21%	73.44%
Random Forest	83.98%	66.10%	73.97%

As can be observed, the threshold *τ *does not affect very much the resulting performance. Although it is to be expected to have fewer confident classifications as *τ *increases, this does not happen. This can be explained by the low frequency and high variability of causal triggers. Classifications are made with similar levels of confidence, regardless of the amount of training data. However, the more training data is given, the more correct classifications are made.

Furthermore, the learning time for each loop increases considerably due to the larger amount of data that needs to be processed into a model. The number of learning loops increases significantly in the case where the seed size is very small. Only few instances are classified with a higher-than-*τ *confidence in each loop, thus resulting in a large number of loops. At the other end, when a large amount of data is available as seed, the training time decreases considerably. As the seed size increases, the classifier becomes more and more confident, and thus more and more instances are added to the labelled group at each step.

The best F-scores are obtained when the ratio is the natural ratio. Actually, the closer the ratio is to the natural one, the better the performance. Training a model on an artificially created corpus, that does not reflect the natural balance, will affect its performance in a real-world situation. The model becomes less strict the more balanced the data is, and will thus produce more false positives. In the case of 1:1 ratio, the recall of the model is very high, reaching values of more than 90%. The precision, however, is extremely low, varying between 10% and 20%. As the seed ratio is shifted towards the natural ratio, the precision and the recall become more balanced: precision increases and recall decreases, but with an overall increased F-score. Unfortunately, space restrictions do not allow for the inclusion of these graphs.

### Argument detection

The process of identifying the two arguments of the causal trigger is divided into three steps. In the first step, a classifier is built in order to determine whether the two arguments are positioned in the same sentence or not, based on the trigger. In the second step, two spans representing the arguments are located around the trigger, either in the same sentence or neighbouring sentences, based on the result of the previous step. The last step deals with giving a sense to the newly found causal relation by assigning roles to the two arguments: cause and effect.

#### Argument position identification

For the purpose of feature extraction, the causal triggers in the unlabelled data set are automatically annotated using the best performing model created in the previous section, which is semi-supervised CRFs. Thus, the errors arising from automatic causal trigger recognition are propagated in the present step.

In case the system gets into the blocked state, we use feature P1 that was previously described: a trigger at the beginning of a sentence signals DS arguments, otherwise SS arguments. The rule is applied on the top 5% confident classifications.

Table 8 shows the best performance achieved by each of the six classifiers used. As can be observed, some F-scores achieved are slightly lower than those obtained in the supervised classification reported in [38]. This happens for the JRip, Random Forest and Vote classifiers and is due to two main reasons. First, the noisy data occurring in the unlabelled set confuses classifiers in their decisions. For instance, one erroneously identified causal trigger is the word *DNA *in sentence (4) below.

**Table 8:** Performance of various semi-supervised algorithms in classifying triggers as SS or DS.

Classifier	P	R	F_1_
Mihăilă et al. [38]	94.75%	94.60%	94.65%

Naïve Bayes	93.56%	96.42%	94.97%
SVM	93.50%	94.44%	93.97%
JRip	91.99%	91.57%	91.78%
J48	93.94%	93.00%	93.47%
RandFor	92.65%	90.04%	91.32%
Vote	93.97%	93.97%	93.97%

(4) The Cre-mediated inverted band ( 6.5 kb) is evident in thymus *DNA *(thymoma).

Another reason is the low recall in recognising triggers. Whilst the precision is high, only a limited set of causal triggers are identified, due to data sparseness.

However, the Naïve Bayes, SVM, and J48 classifiers manage to improve both their precision and recall, which leads to an increased F-score for each of them. In fact, the recall of Naïve Bayes increases considerably, by almost 5%, whilst the precision is almost 2% higher. In the case of SVM, the increase is more moderate, of just 1% in the case of precision and 2% in the case of recall. The improvement of J48 is slightly less than that, with just under 1% for precision and 0.2% for recall.

**Figure 7:**
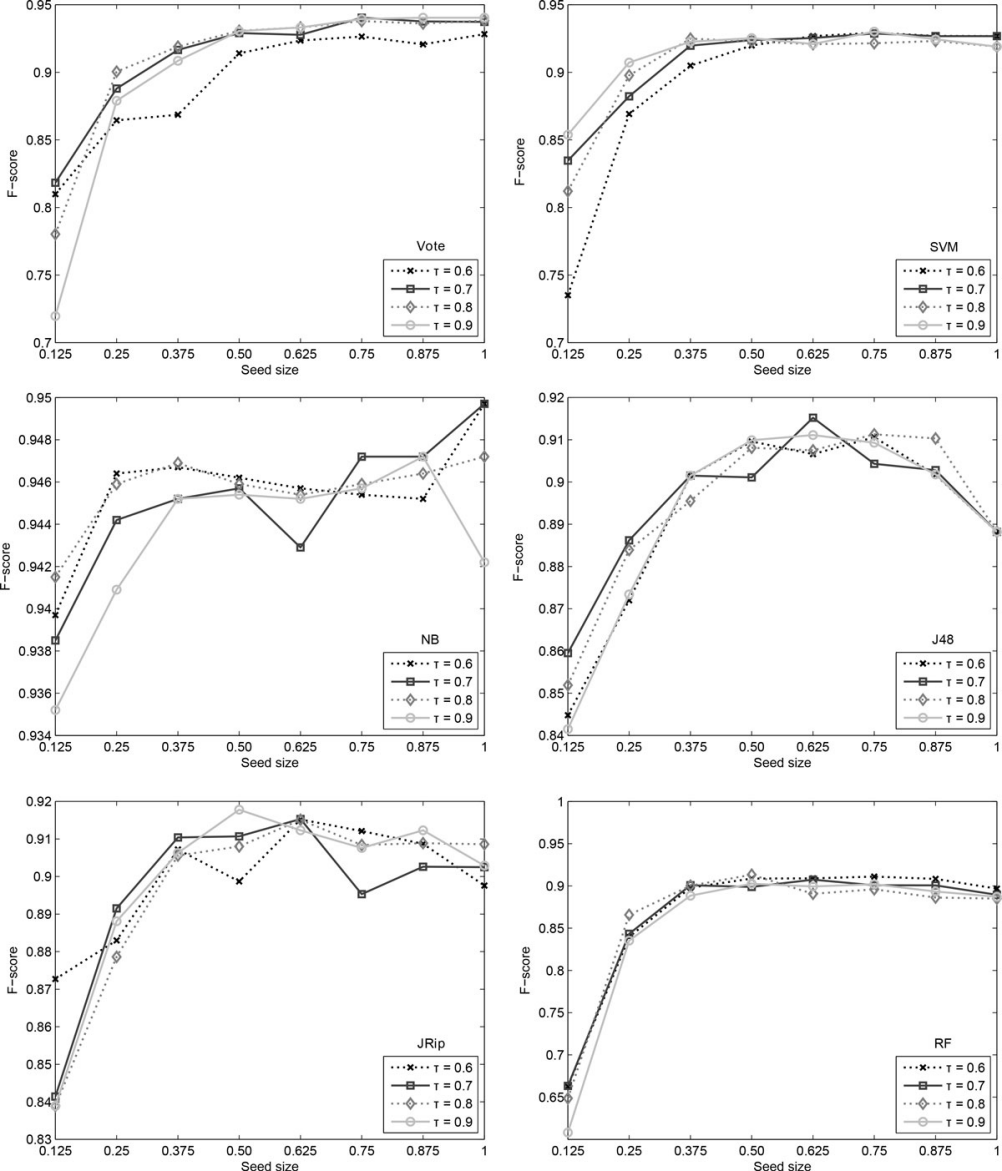
**Argument location results**. Self-training results for the argument location when varying *τ *and the seed size.

We have experimented with various values for the *τ *parameter and the size of the seed data. As before, the *τ *parameter takes values from 0.6 to 0.9, in increments of 0.1, whilst the size of the seed data can vary between 12.5% and 100% in steps of 12.5%. The ratio between positive and negative instances in the seed data has not been included as a parameter, as the data set is roughly balanced. Since the seed data is selected randomly from the labelled set, we repeat each experiment ten times. The average of the obtained results are given for each of the six classifiers in Figure 7.

As can be noticed, the performance of the Naïve Bayes classifier remains relatively insensitive to the variance of both *τ *and seed size. The amplitude of its F-score is just 1.50%, which is not seen in any of the other classifiers. This is partly due to the fact that this specific classifier offers probabilities for each of the two classes that are several orders of magnitude apart. When normalising them, this results in having a binary output, with 0 and 1 as the final probabilities.

The SVM, RF and Vote classifiers suffer significantly when the size of the seed data is 12.50%. All three start at very low values, 61% in the case of RF and 72% in the case of SVM and Vote. The performance quickly increases to over 80% once more data joins the labelled set.

A similar trend is observed on JRip and J48, but to a much lesser degree. In fact, J48 behaves strangely at the other end of the seed size as well. The graph shows a decrease in F-score when 100% of the seed data is available for initial training, which is due to a decrease in precision, whilst the recall remains constant. This happens because of the high variability of low frequency triggers occurring many times non-causally, which allows for the production of many false positives.

The value of the *τ *parameter again does not seem to influence the performance of the classification, especially when more labelled data is available. The only classifier with a visibly separate line for the 60% confidence value for *τ *is Vote. In this case, the performance of the model at 60% confidence threshold is 1-2% lower than the other confidence levels throughout all seed sizes.

#### Argument span identification

The automatic annotations of triggers over the learning data are enhanced with new information regarding the location of the two arguments, obtained from the best performing classifier detailed in the previous section.

Table 9 shows the results that were obtained with the same classifiers as in the case of trigger detection. As can be noticed, CRF leads the performance table, with almost 82% of the arguments identified correctly. SVM and RF are situated at around 5% lower than CRF, whilst NB manages to obtain just 65% F-score.

**Table 9:** Performance of various semi-supervised classifiers in identifying dependent (DA) and independent (IA) argument spans.

Classifier	P	R	F_1_
Mihăilă et al. [38]	74.18%	88.98%	80.91%

CRF	84.52%	79.58%	81.98%
SVM	75.85%	77.95%	76.89%
Random Forest	76.95%	76.50%	76.72%
Naïve Bayes	63.30%	67.35%	65.26%

We have identified several errors arising from the automatic annotation of the unlabelled data by using the models from previous steps. There are several cases in which a same-sentence trigger is erroneously classified as different-sentence, such as the one in example (5). This type of errors is due to the order of the causal constituents, T-E-C in this case. Since the trigger is the first token in the sentence, the algorithm decides that the arguments are located in distinct sentences.

(5) *Since *[Brucella is an intracellular facultative pathogen]*_DA_*, [the bacteria could use these denitrification reactions to grow under low-oxygen condition by respiration of nitrate]*_IA_*.

The reverse occurs as well: there are several cases where different-sentence triggers are classified as being same-sentence, as shown in example (6). This happens when the trigger is located mid-sentence and the majority of its occurrences are in fact same-sentence.

(6) [The fact that PmrB is likely to sense changes in pH directly]*_DA _is supported by *multiple findings. First, [the mild acid pH-dependent activation of the PmrA-regulated gene pbgP was dramatically reduced in a strain lacking pmrB]*_IA_*.

**Figure 8:**
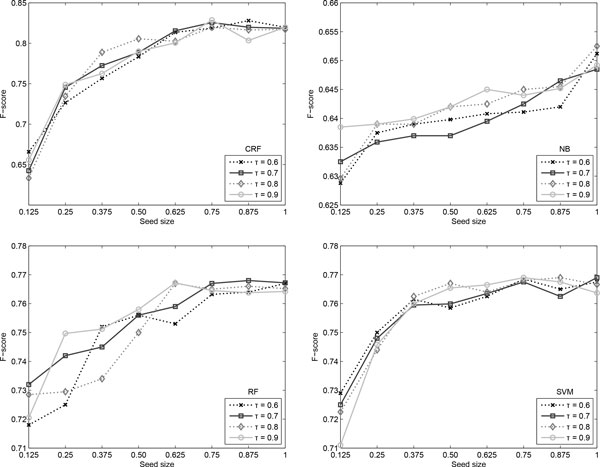
**Argument span results**. Self-training results for the argument span identification when varying *τ *and the seed size.

Figure 8 depicts the change in the obtained F-score when varying the seed size and confidence threshold for each of the four classifiers. As noticed before, the Naïve Bayes classifier has a very small amplitude in the F-score curve, of just over 2%. In contrast, the other three algorithms increase their performance by approximately 5% when changing the size of the seed data from 12.5% to 100%. All classifiers are, however, insensitive to the modification of the confidence threshold, especially when higher amounts of seed data are available.

#### Argument role identification

The final step in the causality recognition pipeline is to detect which argument plays which semantic role. Each of the previously identified arguments must be assigned one of the two possible roles, Cause and Effect. For this task, we have explored different possibilities to detect whether a causal relation is of the form C-T-E or E-T-C. The other three possibilities existing in BioCause have been excluded from the classification, as their number is insufficient for training purposes. One aspect that has to be taken into consideration is the skewed data, which has a ratio E-T-C to C-T-E of 1:7.54. In addition, the argument spans are automatically detected using the best performing classifier described in the previous step.

Table 10 lists the results obtained by the six classifiers used as learning algorithms. The Vote meta-classifier has obtained the best performance, an F-score of 83.79%. However, it is slightly lower than that obtained in a supervised setting by Mihăilă et al. [38]. This is due to the propagation of errors from the previous two steps.

**Table 10:** Performance of various semi-supervised classifiers in identifying argument roles.

Classifier	P	R	F_1_
Mihăilă et al. [38]	85.25%	83.55%	84.35%

Naïve Bayes	70.45%	80.05%	74.94%
SVM	82.50%	80.05%	81.25%
JRip	84.65%	80.90%	82.73%
J48	83.10%	79.20%	81.10%
RandFor	79.85%	74.20%	76.92%
Vote	84.55%	83.05%	83.79%

Besides the errors regarding the classification of the trigger into SS or DS, exemplified in the previous section, the current step inherited inaccurate spans for the arguments. Most common is the case of selecting the wrong span for the arguments located in a different sentence by choosing a completely wrong sentence. Another possibility is only the partial match for an argument, where the classifier also selects false positives and leaves out false negatives.

**Figure 9:**
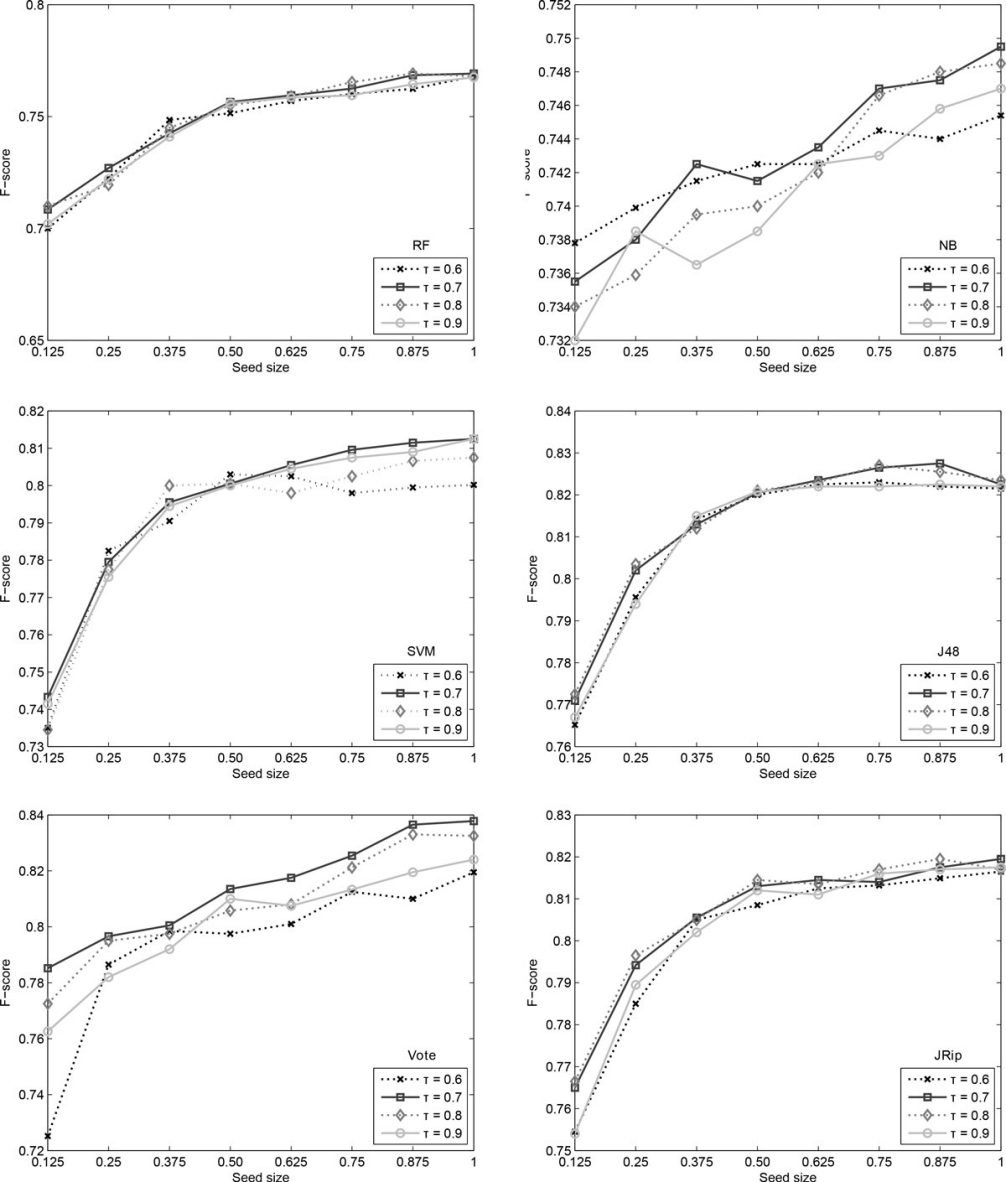
**Argument role results**. Self-training results for the argument role identification when varying *τ *and the seed size.

Figure 9 shows the variation in F-score when changing the seed size and confidence threshold. As can be noticed, most classifiers have a generally increasing trend, with a high slope for small amounts of seed data. As this size increases, the slope of the F-score curve decreases and almost plateaus towards 100% of the seed data. Naïve Bayes is, in contrast to all other classifiers, fairly constant throughout different seed sizes. However, its performance is the worst, at almost 10% distance from Vote.

The confidence threshold *τ *does not generally influence the performance of the algorithms. Notable cases are the value of 60% confidence, which obtains a low F-score for the Vote classifier at seed size 12.5% and for SVM at high seed sizes.

## Discussion

There are two major factors to be considered when automatically recognising triggers and their arguments: the chosen algorithm and the selection of features. They are all discussed in the following sections.

### Trigger detection

The experiments performed and discussed in the previous sections show that a semi-supervised approach yields a better F-score. More specifically, employing a supervised CRFs reaches an F-score of 79.00%, whilst RFs and SVM perform worse by around 8%. On the other hand, a semi-supervised approach produces higher results. If the learning is performed on unlabelled data, the performance increases to 83.35% in the case of CRFs, and to almost 74% in the case of RFs and SVMs.

These results are much lower than those that are obtained in the open domain. Pitler et al. [11], for instance, achieve results as high as 91% F-score using Naïve Bayes on automatic parses when identifying discourse triggers in general, whilst Lin et al. [12] obtain 93.62% F-score. Ibn Faiz et al. [18] further improve the results to 96.22% F-score. However, assigning senses to the relations seems to be more difficult. The F-score of Lin et al. [12] reaches only 80%, whilst Pitler et al. [11] perform a level 1 type sense assignment and obtain 94% F-score. In the level 1 type classification, Causality is part of the Contingency class, together with Pragmatic Cause, Condition and Pragmatic Condition. Thus, if we consider these two steps as leading to the same goal as our task, then by multiplying the two results (93.62% and 80%) we get a performance of around 75%, less than the one described in this chapter. Nevertheless, when applying a model trained on BioDRB on the PDTB corpus, similar results are obtained [17]. This shows that in-domain classifiers outperform cross-domain classifiers and that biomedical scientific discourse is truly different and more difficult to capture automatically.

Both CRFs and SVMs have been used before in detecting biomedical discourse triggers, although they have not been trained on causality specifically. Ramesh et al. [17] experimented with these two algorithms on BioDRB, and concluded that the CRF model outperformed the SVM model by 10%, producing a final F-score of 75.70%. More recently, the same corpus has been used by Ibn Faiz et al. [18], who applied their extended feature set with ME classifiers and achieved a performance of 82.36% F-score. Again, they make no distinction between the various discourse relations and treat them as a whole.

However, the RF algorithm has not been used before for this task.

In what regards semi-supervised approaches, the literature is not very vast, and does not contain any work on biomedical data. Our self-training method is, to the best of our knowledge, the first semi-supervised approach of this type applied to discourse connective recognition. A different approach is that of Hernault et al. [39], who prove that feature vector extension is a promising method to improve classification accuracy for infrequent discourse relation types. Evaluating it on PDTB, the method increases the baseline F-score by more than three times in some cases for discourse causality, to 18.7%. However, as the authors themselves admit, this method cannot be used by itself in discourse analysis due to its low performance.

Do et al. [40] develop a minimally supervised event causality identification methodology, which employs a measure of cause-effect association between two given events and their arguments. They obtain an F-score of 38.60% on PDTB, but this increases to 41.70% when joint inference is performed with discourse relation predictions from inductive logic programming.

In what concerns features, we noticed through our experiments that the best performance is obtained when using all types of features. This includes domain independent features, such as syntactic, dependency and command features, but also domain specific features, such as biomedical semantics. In fact, semantics plays a very significant role in the task of recognising causal triggers. They improve the classification in most feature combinations, and increase the performance by 2.13% on average.

On biomedical text, Ramesh et al. [17] employs mostly orthographic features and just a few syntactic features. They also include named entity information obtained from UMLS and ABNER, but conclude that it damages the overall performance. More specifically, the F-score drops with between 1% and 7.5%, depending on the semantic feature source. In their case, recall is most affected, with variations of even 10%, whilst precision is relatively constant, but still falling with up to 3%. Ibn Faiz et al. [18] suggest that the reason behind semantics damaging the performance of Ramesh et al. [17] is the fact that ABNER already uses orthographic features, which thus get duplicated in the feature vector.

As Ibn Faiz et al. [18] also suggest in their error analysis, there are cases of discourse triggers which cannot be captured by using only surface level and syntactic features, and instead need some sort of semantic understanding of the context. By checking the children of the dominant SBAR of the trigger for temporal senses, they manage to slightly increase the performance with 0.18%. Our richer semantic features add much more than that.

In conclusion, all feature types are needed and complement each other. Whilst lexical features are the most indicative of causal triggers, syntax and semantics permit generalisation over the grammatical flexibility and sense variability of language.

Having compared our results to the current state-of-the-art, we consider our supervised and semi-supervised CRFs to improve on it in biomedical discourse causal trigger recognition. The main result of this experiment is the fact that more data is needed for such specialised domains.

### Argument detection

Our experiments have shown that causal arguments are best detected in a supervised setting. This is due to the fact that the errors occurring in previous steps are propagated and affect the performance of semi-supervised systems. Nevertheless, the performance between the supervised and semi-supervised in comparable, even with error propagation.

For the first and third steps, we employed six different classifiers, one of them making its decisions based on the result of the other five. The wide spectrum of algorithms, ranging from Naïve Bayes to decision rules, decision trees and SVMs, provide complementary results which lead the Vote meta-classifier to outperform them by up to 2% for the first step and 3% for the third step.

For the second step, we modelled the task as a sequence labelling task using CRFs, and as a classification task using SVMs, RFs and NB. CRF performed best in this case, surpassing SVM and RF by approximately 5%, and NB by 16%.

The literature is very restricted from this point of view: most research is either based on CRFs, when researchers perform a token-level identification [41,42], or on ME classifiers when they wish to obtain syntactic constituents that span the arguments [12,43].

With respect to features, in all the experiments that we described, using features from all types produced the best results. This includes both domain-independent features, such as lexical, syntactic and positional features, and features specific to the biomedical domain, such as biomedical semantics. Semantics has proven to play a major role especially in the argument span and role recognition, where they improve the F-score by 3% on average.

The task of detecting the arguments of causal relations, and, more generally, discourse relations, has not been as studied as recognising triggers. Thus, the variety of features that have been employed until now is fairly limited. Do et al. [40] use a complex semantic feature measuring the similarity between two predicates, including their arguments, in the general domain, for the task of deciding whether or not the pair of predicates are in a causal relation. Their method takes into consideration just co-occurrence and various distances between the two predicates, but it manages to improve the F-score by 15% over that obtained by classical point-wise mutual information, to 38%. It is recall that is increased significantly in this case, from 26% to 62%, when tested on PDTB.

Other methods restrict themselves to lexical and syntactic features. Ghosh et al. [26], Lin et al. [12] and Xu et al. [43] engineer a similar feature set to each other in their own approaches. Whilst Ghosh et al. [26] uses a features set composed of lexical features (surface expression and lemmata of tokens) and morpho-syntactic features (PoS, inflection, main verb of sentence, path from root to token in parse tree), Lin et al. [12] extends it by adding information about the neighbouring tokens. Xu et al. [43] enriches the set even more, considering the position of the token relative to the trigger (left or right), and its position in the sentence as a binary class (before the middle or after the middle of the sentence). Thus, they manage to reach 46% F-score in recognising both arguments when they employ automatic parses for feature extraction.

On biomedical text, the relevant literature is extremely limited. To the best of our knowledge, Ibn Faiz et al. [18] describe the only method that identifies argument head words in the style of Wellner et al. [27]. However, no decision is made on argument spans. To note is the fact that their system has been built having the general domain in mind, and just applied on biomedical data. Thus, the framework does not use biomedical-specific processing or features specific to the biomedical domain.

In conclusion, all feature types are needed for a better performance in discourse argument identification, as they complement each other. Whilst lexical and positional features increase precision, semantic and syntactic information boost recall.

## Conclusions

This article has described our three-step approach to automatically recognise causal relations in biomedical scientific discourse in a semi-supervised learning setting. We augment the BioCause corpus, containing gold standard causal relation annotations, with existing unlabelled data. Furthermore, we add new structural features, regarding c-command relations in parse trees, and positional features, which can reduce the number of false positives and negatives.

Having access to more data, semi-supervised machine learners improve their performance over supervised in the first three steps, even when the errors propagate through the pipeline. Trigger spans are recognised with a 4.35%-increased F-score. The position of arguments and their spans also benefit from unlabelled data, with increases in F-score of 0.32% and 1.07%, respectively. In the last step, which assigns roles to arguments, the top F-score is 0.56% lower than that reported in a supervised setting by Mihăilă et al. [38].

Feature-wise, the performance of this step might be improved by the addition of a causality measure that can capture the uni-directionality of this type of discourse relation. Data-wise, we emphasise the acute need of more gold-standard annotations in order to better capture and represent the variety and ambiguity of language, both in the seed and test datasets.

## Competing interests

The authors declare that they have no competing interests.

## Authors' contributions

All authors contributed to the production of the manuscript. SA supervised all steps of the work. CM designed and performed the experiments and analysed the results. All authors read and approved the final manuscript.

## Declarations

Publication of this article has been funded by the EPSRC [grant number EP/P505631/1]; Medical Research Council; Europe PubMed Central (led by Wellcome Trust).

This article has been published as part of *BioMedical Engineering OnLine *Volume 13 Supplement 2, 2014: Selected articles from the IEEE International Conference on Bioinformatics and Biomedicine (BIBM 2013): BioMedical Engineering OnLine. The full contents of the supplement are available online at http://www.biomedical-engineering-online.com/supplements/13/S2.
